# Nutritional and antioxidant components and antioxidant capacity in green morph *Amaranthus* leafy vegetable

**DOI:** 10.1038/s41598-020-57687-3

**Published:** 2020-01-28

**Authors:** Umakanta Sarker, Md. Motaher Hossain, Shinya Oba

**Affiliations:** 1 Department of Genetics and Plant Breeding, Faculty of Agriculture, Bangabandhu Sheikh Mujibur Rahman Agricultural University, Gazipur, 1706 Bangladesh; 2 Department of Plant Pathology, Faculty of Agriculture, Bangabandhu Sheikh Mujibur Rahman Agricultural University, Gazipur, 1706 Bangladesh; 3 Laboratory of Field Science, Faculty of Applied Biological Sciences, Gifu University, Yanagido 1-1, Gifu, Japan

**Keywords:** Biochemistry, Plant sciences

## Abstract

Amaranth has two morphological types described as red and green morphs. Previous studies have extensively characterised red morph amaranth regarding both morphological and chemical properties including antioxidant activity, antioxidant phytochemical profile, mineral content and proximate composition. However, there is scarce information concerning green morph amaranth. Hence, the present study evaluated 12 green morph genotypes for proximate composition, antioxidant activity, antioxidant pigments, minerals, and phytochemicals. Green morph amaranth was found to contain abundant carbohydrates, dietary fiber and protein. We found notable levels of inorganic minerals including potassium, calcium, magnesium, iron, manganese, copper and zinc. Antioxidant capacity quantified as free radical quenching capacity varied between 27 and 48 μg g^−1^ Trolox equivalents. We additionally quantified antioxidants, including total phenolics, total flavonoid equivalents and vitamin C, as well as the antioxidant pigments carotenoids, chlorophylls and betalains. These data indicated that four green morph genotypes could be considered as enriched in their antioxidant profiles. Green morph amaranth could be a potential source of nutritional components and antioxidant phytochemicals in the human diet providing opportunities to address mineral nutrient deficiencies and provide an antioxidant rich food

## Introduction

*Amaranthus* is a fast-growing plant that is widely distributed throughout the world and belongs to the family Amaranthaceae. Among the 70 species of *Amaranthus*, 17 are cultivated for edible leaves, and 3 are cultivated as food grains^1^. A few genera of *Amaranthus* are used as a traditional medicine for the remedy of viral, malarial, diabetic, bacterial, and helminthic diseases and as snake bite antidote^2–4^. Amaranth leaves and stems are good economic sources of carotenoids, proteins, including the essential amino acids methionine and lysine, dietary fiber and minerals, such as magnesium, calcium, potassium, copper, phosphorus, zinc, iron, and manganese^5–16^. Amaranth is also abundant in several pigments, such as carotenoids, chlorophylls, amaranthine, anthocyanins, betalains, betaxanthins, and betacyanins^17,18^ and natural antioxidant phytochemicals, such as vitamin C, betacarotene, flavonoids, and phenolic acids^19,20^, that act as reactive oxygen species (ROS) scavengers in the human body^21,22^. This plant family is widely acclimatized to different abiotic stresses, such as drought^23–26^ and salinity^27–29^, and has multipurpose applications.

Amaranth has two morphological types described as red and green morphs^30^. Bangladesh has many amaranth germplasms with great variability and phenotypic plasticity^31^ that have many culinary uses. The edible green morph of amaranth is a famous vegetable in Bangladesh, South-East Asia, and Africa. Its nutritional value, taste, and attractive leaf color make it very popular. In Bangladesh, amaranth is grown year-round and even fills in the gaps of main crops between winter and summer^5,6^.

Currently, food scarcity results in a continuous calorie deficit of approximately 795 million malnourished people^32^. Vitamin or mineral deficiencies can cause hidden hunger in over two billion people^33^. As a source of energy, staple foods are deficient in micronutrients, consequently resulting in hidden hunger^33,34^. Therefore, a good way to ensure a healthy and balanced diet by consuming vegetables and fruit as a source of vitamins and minerals along with staple foods. Furthermore, we can protect human health and reduce the risk of cancer, cardiovascular disease, and other chronic diseases by consuming fruits and vegetables. Phytochemical compounds, such as phenolics, flavonoids, vitamin C, and leaf pigments contribute to numerous health benefits^21,35,36^.

Recently, the natural antioxidants present in vegetables have gained the attention of consumers and researchers. Amaranth contains abundant natural antioxidants, such as flavonoids, pigments, phenolics, carotenoids, and vitamin C^21,22^. These natural antioxidant phytochemicals defend against several diseases, such as cardiovascular diseases, cancer, cataracts, atherosclerosis, retinopathy, arthritis, emphysema, and neurodegenerative diseases^21,35,36^.

Although Amaranth is a low-cost source of minerals, dietary fiber, protein, and antioxidant compounds, the information on its contents is scarce. In our earlier study, we evaluated the red morph of amaranth in terms of its morphological characteristics and mineral, protein, dietary fiber, betacarotene, and ascorbic acid contents^5–9^. However, to our knowledge so far, there is no acceptable and reliable information on the proximate and mineral compositions and the vitamin, phenolic, and flavonoid contents in the large number of diversified green morphs of amaranth germplasms available in Bangladesh and elsewhere.

Therefore, to fill these gaps, we carried out this study with the following aims:

1. To evaluate the proximate and mineral compositions and the vitamin, phenolic, and flavonoid contents in 12 green morphs of amaranth genotypes.
2. To determine the variability of these traits in 12 green morphs of amaranth genotypes.

## Results and Discussion

From the analysis of variance, it was demonstrated that all of the studied traits differed significantly among the genotypes.

### Proximate compositions

Table 1 outlines the proximate compositions of the green morph amaranth. GRA15 exhibited the highest moisture content (884.54 g kg^−1^ FW), while GRA12 had the lowest moisture content (814.83 g kg^−1^ FW). The moisture content ranged from 814.83 to 884.54 g kg^−1^ FW. As leaf dry matter increased with a lower moisture content, six genotypes, including GRA12, GRA7, GRA17, and GRA9, had desirable dry matter contents (18–19%). The moisture content of the green morph amaranth leaves was directly related to the maturity of the plant. The results obtained in this study were similar to those of red amaranth and sweet potato leaves by Sarker and Oba^23^ and Sun *et al*.^37^, respectively.

**Table 1:** Proximate compositions (g kg^−1^ fresh weight) and dietary fiber (g 100 g^−1^ FW) in green morph *Amaranthus* leafy vegetable.

Genotypes	Moisture(g kg^−1^)	Protein(g kg^−1^)	Fat (g kg^−1^)	Carbohydrates(g kg^−1^)	Energy (Kcal)	Ash (g kg^−1^)	Dietary fiber(g 100 g^−1^ FW)
GRA3	878.22 ± 3.85	36.31 ± 1.18	2.42 ± 0.04	54.21 ± 1.82	36.05 ± 2.07	28.84 ± 1.17	7.83 ± 0.65
GRA4	866.62 ± 4.16	33.72 ± 0.97	2.62 ± 0.03	74.46 ± 1.74	44.56 ± 2.34	22.58 ± 1.24	8.21 ± 0.31
GRA7	821.58 ± 5.34	34.35 ± 1.17	2.31 ± 0.03	90.21 ± 1.77	52.99 ± 2.26	51.55 ± 1.16	7.82 ± 0.64
GRA9	837.49 ± 6.53	33.38 ± 2.15	2.68 ± 0.04	80.19 ± 1.12	46.61 ± 1.66	46.26 ± 0.97	9.55 ± 0.85
GRA11	854.55 ± 5.32	38.13 ± 1.56	2.78 ± 0.02	70.08 ± 1.16	41.72 ± 2.09	34.46 ± 1.08	6.02 ± 0.28
GRA12	814.83 ± 5.14	50.99 ± 1.64	2.86 ± 0.01	80.09 ± 1.22	56.07 ± 2.33	51.23 ± 0.84	6.96 ± 0.23
GRA15	884.54 ± 4.86	54.98 ± 0.98	2.58 ± 0.03	15.48 ± 1.38	26.95 ± 2.71	52.38 ± 1.15	7.73 ± 0.16
GRA17	835.12 ± 3.72	40.82 ± 0.75	4.21 ± 0.02	72.87 ± 1.65	45.14 ± 1.24	46.85 ± 0.86	7.21 ± 0.34
GRA21	869.74 ± 5.12	44.62 ± 1.22	3.15 ± 0.03	66.96 ± 1.26	36.27 ± 2.18	33.43 ± 1.08	6.68 ± 0.65
GRA23	847.68 ± 2.74	35.65 ± 1.86	4.14 ± 0.03	75.52 ± 1.18	40.15 ± 2.35	45.57 ± 0.96	6.23 ± 0.03
GRA26	864.92 ± 4.38	23.87 ± 1.18	3.45 ± 0.01	57.49 ± 1.15	40.27 ± 2.43	32.24 ± 1.04	8.56 ± 0.37
GRA29	880.78 ± 3.36	37.92 ± 1.12	3.24 ± 0.02	54,21 ± 1.78	39.78 ± 3.15	20.57 ± 1.12	7.35 ± 0.88
Grand mean	854.68	38.73	3.06	67.05	42.21	38.83	7.51
F values	**	**	**	**	**	**	**
SE	1.517	0.515	0.012	1.245	0.716	1.222	0.178
CV%	2.226	1.248	0.227	0.907	0.723	0.682	0.218
CD	4.276	1.452	0.034	3.510	2.018	3.445	0.502

SE, Standard error difference; CV, Coefficient of variation; CD, Critical difference; n = 3; **Significant at 1% level.

Green morph amaranth leaves exhibited clear variations in protein content. The GRA15 genotype had the highest protein content (54.98 g kg^−1^) followed by that of GRA12, whereas GRA26 exhibited the lowest protein content (23.11 g kg^−1^). Four genotypes performed better than the mean protein content across all genotypes. The genotypes GRA12, GRA15, GRA17, and GRA21 had relatively high protein contents for a leafy vegetable. Vegetarians and many people in low-income countries mainly depend on amaranth for their protein source. The protein content of the green morph amaranth (38.73 g kg^−1^) was much higher than that of the red morph amaranth (1.26%) determine in our earlier study^8^. The fat content was the highest in GRA17 (4.21 g kg^−1^ FW) with the order of GRA17 > GRA23 > GRA26 GRA29 > GRA21. The lowest fat content was found in GRA7 (2.31 g kg^−1^ FW), with an average of 3.06 g kg^−1^ FW. Sarker and Oba^23^ and Sun *et al*.^37^ observed similar results in *A. tricolor* and sweet potato leaves, respectively. It is known that fat is involved in the insulation of body organs and the maintenance of proper body temperature and cell function. Fats have abundant omega-3 and omega-6 fatty acids and play a significant role in absorption, digestion, and transport of fat-soluble vitamins A, D, E, and K.

GRA7 had the highest carbohydrate content (90.21 g kg^−1^ FW), followed by that of GRA9 and GRA12; moreover, the lowest carbohydrate content was recorded in GRA15 (15.48 g kg^−1^ FW), with an average content of 67.05 g kg^−1^ FW. The genotype GRA12 had the highest energy (56.07 kcal 100 g^−1^ FW), followed that of by GRA7 and GRA17, while the lowest energy was observed in genotype GRA15 (26.95 kcal 100 g^−1^ FW), with an average of 42.21 kcal 100 g^−1^ FW. The ash content was the highest in GRA15 (52.38 g kg^−1^ FW), followed by that in GRA12 and GRA7, and the lowest ash content was noted in GRA29 (20.57 g kg^−1^ FW), with an average of 38.83 g kg^−1^ FW.

The dietary fiber content exhibited significant variations among the amaranths studied. Dietary fiber was highest in GRA9 (9.55 g 100 g^−1^ FW), followed by that in GRA26 and GRA4, where the lowest was in GRA11 (6.02 g 100 g^−1^ FW), with an average of 7.51 g 100 g^−1^ FW. Dietary fiber significantly contributes to mitigating constipation and to food digestibility and palatability^5^. From the results, we observed that the green morph amaranth leaves have abundant carbohydrates, protein, moisture, and dietary fiber. The results of this study corroborated the results of Sarker and Oba^23^ regarding red amaranth.

### Mineral compositions

The mineral compositions of the green morphs of amaranth are shown in Table 2. Our study revealed that the K content ranged from 4.19 mg g^−1^ to 5.17 mg g^−1^ FW. The genotypes GRA23, GRA9, GRA11, GRA3, GRA12, and GRA15 had a high K content, while genotype GRA21 showed the lowest, with an average of 4.91 mg g^−1^ FW. Seven genotypes exhibited K contents that were greater than the average K content. The calcium content ranged from 1.89 to 3.09 mg g^−1^ FW. The genotypes GRA4, GRA12, GRA29, GRA23, and GRA11 showed a high Ca content, and GRA7 had the lowest Ca content, with an average of 2.14 mg g^−1^ FW. Six genotypes had a higher Ca content than the average Ca content. The Mg content was the highest in GRA23 and GRA4, while the lowest was recorded in GRA17, with an average of 2.92 mg g^−1^ FW. The genotypes GRA4, GRA23, GRA3, GRA26, GRA21, GRA15, and GRA12 had relatively high Mg contents. In this study, the genotypes did not show considerable variations in terms of Mg content (2.84 to 3.01 mg g^−1^ FW). In our present study, we found noteworthy quantities of K (4.91 mg g^−1^), Ca (2.57 mg g^−1^) and Mg (2.92 mg g^−1^) in the green morph amaranth (fresh weight basis). Jimenez-Aguiar and Grusak^38^ found abundant K, Ca and Mg contents (fresh weight basis) in different *A. spp*. They concluded that in amaranth, the K, Ca, and Mg contents were much higher than in the spider flower, black nightshade, spinach, and kale. Our studied green morph amaranth had higher K, Ca, and Mg contents (fresh weight basis) compared to those of the studied *A. spp* reported by Jimenez-Aguiar and Grusak^38^.

**Table 2:** Mineral compositions (Macro mg g^−1^ FW and micro µg g^−1^ FW elements) in green morph *Amaranthus* leafy vegetable.

Genotypes	Macroelements (mg g^−1^ FW)			Microelements (µg g^−1^ FW)			
	K	Ca	Mg	Fe	Mn	Cu	Zn
GRA3	5.02 ± 0.15	2.62 ± 0.29	2.97 ± 0.11	17.83 ± 0.67	11.34 ± 0.12	1.29 ± 0.03	17.03 ± 0.12
GRA4	4.85 ± 0.12	3.09 ± 0.16	3.00 ± 0.04	11.17 ± 0.38	9.26 ± 0.11	2.16 ± 0.02	14.00 ± 0.18
GRA7	4.98 ± 0.18	2.14 ± 0.27	2.91 ± 0.12	9.80 ± 0.25	12.78 ± 0.11	1.26 ± 0.03	10.40 ± 0.15
GRA9	5.13 ± 0.12	1.89 ± 0.16	2.84 ± 0.13	7.39 ± 0.92	8.76 ± 0.18	1.24 ± 0.07	10.00 ± 0.01
GRA11	5.07 ± 0.08	2.79 ± 0.12	2.85 ± 0.15	14.87 ± 0.09	14.75 ± 0.12	3.18 ± 0.04	9.50 ± 0.16
GRA12	5.00 ± 0.15	3.09 ± 0.11	2.94 ± 0.12	10.97 ± 0.27	15,89 ± 0.17	1.24 ± 0.01	9.87 ± 0.14
GRA15	5.00 ± 0.21	2.39 ± 0,14	2.95 ± 0.09	10.25 ± 0.28	11.88 ± 0.16	1,26 ± 0.02	9.43 ± 0.17
GRA17	4.76 ± 0.13	2.29 ± 0.15	2.83 ± 0.06	9.25 ± 0.58	7.48 ± 0.18	1.25 ± 0.06	9.01 ± 0.18
GRA21	4.19 ± 0.24	2.38 ± 0.12	2.94 ± 0.13	7.86 ± 0.25	5.82 ± 0.15	1.27 ± 0.07	8.81 ± 0.15
GRA23	5.17 ± 0.15	2.79 ± 0.12	3.01 ± 0.17	9.63 ± 0.47	13,75 ± 0.19	1.28 ± 0.01	9.81 ± 0.19
GRA26	4.86 ± 0.09	2.47 ± 0.18	2,97 ± 0.15	9.05 ± 0.38	12.87 ± 0.22	1.30 ± 0.04	9.80 ± 0.18
GRA29	4.88 ± 0.12	2,94 ± 0.05	2.90 ± 0.08	13.74 ± 0.27	15.34 ± 0.16	1.18 ± 0.09	12.01 ± 0.09
Grand mean	4.91	2.57	2.92	10.98	11.66	1.49	10.81
F values	**	**	**	**	**	**	**
SE	0.012	0.021	0.024	0.035	0.021	0.043	0.062
CV%	0.122	0.352	0.155	0.286	0.384	0.122	0.242
CD	0.034	0.059	0.068	0.099	0.059	0.121	0.175

SE, Standard error difference; CV, Coefficient of variation; CD, Critical difference; K, Potassium; Ca. Calcium, Mg, Magnesium; Fe, Iron; Mn, Manganese; Cu, Copper; Zn, Zinc, n = 3; **Significant at 1% level.

The iron content showed significant and remarkable variations (9.25 µg g^−1^ FW in GRA17 to 17.83 µg g^−1^ FW in GRA3) among the genotypes. The genotypes GRA3, GRA4, GRA11, GRA12, and GRA15 had the highest iron contents. Conversely, the genotype GRA9 showed the lowest iron content, with an average value of 10.98 µg g^−1^ FW. Four genotypes had higher iron contents than the average Fe content.

In this study, the manganese content ranged between 5.82 µg g^−1^ FW and 15.89 µg g^−1^ FW, with an average of 11.66 µg g^−1^ FW. The genotypes GRA12, GRA29, GRA11, GRA23, GRA26, GRA15, GRA7, and GRA3 had high manganese contents; however, the genotype GRA21 exhibited the lowest manganese content (5.82 µg g^−1^ FW). Additionally, the genotypes showed significant and notable variations in copper content (1.18–3.18 µg g^−1^ FW). The copper content was the highest in GRA11 (3.18 µg g^−1^ FW), followed by that in GRA4. Two genotypes exhibited a higher copper content that the corresponding mean of all samples. The genotypes differed significantly and remarkably in terms of zinc content (8.81 µg g^−1^ FW (GRA21) to 17.03 µg g^−1^ FW (GRA3). Three genotypes showed higher zinc contents that the mean content (10.81 µg g^−1^ FW). The green morph amaranth has higher zinc and iron contents compared to those of cassava leaves^39^ and beach pea^40^. In this study, we found remarkable Fe (10.89 µg g^−1^), Mn (11.66 µg g^−1^), Cu (1.49 µg g^−1^) and Zn (10.81 µg g^−1^) contents in the green morph amaranth (fresh weight basis). Jimenez-Aguiar and Grusak^38^ noted abundant Fe, Mn, Cu, and Zn contents in different amaranths, including red amaranth. They also found that in amaranth Fe, Mn, Cu, and Zn were much higher than in spinach, spider flower, kale, and black nightshade.

### Antioxidant pigments

The antioxidant pigments detected in the green morph amaranth are presented in Table 3. The chlorophyll *a* content (126.47 to 429.62 μg g^−1^ FW) exhibited noticeable variations among the genotypes. The highest chlorophyll *a* content (429.62 μg g^−1^ FW) was recorded in the genotype GRA4, while GRA15 showed the lowest chlorophyll *a* content (126.47 μg g^−1^ FW). The genotypes GRA9 and GRA23 had relatively high chlorophyll *a* contents. Five genotypes exhibited a higher chlorophyll *a* content than the mean content of all genotypes. Like chlorophyll *a*, significant and marked differences were observed in the chlorophyll *b* content (50.72 to 239.09 μg g^−1^ FW) as well. The highest chlorophyll *b* content (239.09 μg g^−1^ FW) was recorded in GRA4, followed by that in GRA21 and GRA23. In contrast, the genotype GRA29 exhibited the lowest chlorophyll *b* content (50.72 μg g^−1^ FW). Chlorophyll *ab* showed significant variations (179.94 to 669.72 μg g^−1^ FW) among the genotypes. The genotype GRA23, GRA21, GRA9, and GRA7 exhibited high chlorophyll *ab* contents, whereas the lowest was recorded in genotype GRA4 (179.94 μg g^−1^ FW). Two genotypes had higher chlorophyll *ab* contents than the mean value. In this study, we found good chlorophyll *ab* (555.24 μg g^−1^ FW), chlorophyll *b* (115.14 μg g^−1^ FW), and chlorophyll *a* (239.10 μg g^−1^ FW) contents in the green morphs of amaranth, whereas Khanam and Oba^40^ observed comparatively low chlorophyll contents in red morphs of amaranth.

**Table 3:** Mean performance for antioxidant leaf pigments in green morph *Amaranthus* leafy vegetable.

Genotypes	chlorophyll *a*(μg g^−1^ FW)	Chlorophyll *b* (μg g^−1^ FW)	Chlorophyll *ab* (μg g^−1^ FW)	Betacyanin (ng g^−1^ FW)	Betaxanthin (ng g^−1^ FW)	Betalain (ng g^−1^ FW)	Carotenoids(mg 100 g^−1^ FW)
GRA3	127.26 ± 2.17	51.67 ± 0.88	179.94 ± 1.09	240.28 ± 0.71	144.11 ± 0.61	384.39 ± 1.12	23.02 ± 0.25
GRA4	429.62 ± 3.12	239.09 ± 0.77	669.72 ± 2.12	238.51 ± 0.68	154.31 ± 0.55	392.82 ± 1.24	35.53 ± 0.24
GRA7	254.38 ± 2.11	93.33 ± 0.85	348.72 ± 3.03	128.75 ± 0.81	152.75 ± 0.74	281.50 ± 2.28	75.03 ± 0.46
GRA9	360.71 ± 3.24	120.97 ± 0.66	482.69 ± 2.15	152.26 ± 0.56	164.29 ± 0.68	316.55 ± 1.15	13.72 ± 0.47
GRA11	172.75 ± 4.17	97.55 ± 0.98	271.31 ± 2.11	94.51 ± 0.39	79.40 ± 0.74	173.91 ± 2.18	14.16 ± 0.48
GRA12	200.52 ± 2.14	91.90 ± 0.75	293.44 ± 3.11	156.25 ± 0.54	85.48 ± 0.53	241.73 ± 1.24	48.16 ± 0.66
GRA15	126.47 ± 1.11	65.83 ± 0.65	193.31 ± 1.21	138.51 ± 0.81	146.3 ± 0.41	284.81 ± 1.24	89.44 ± 0.39
GRA17	221.61 ± 2.07	100.62 ± 0.57	323.24 ± 2.12	146.05 ± 0.57	75.32 ± 0.17	221.37 ± 2.16	27.52 ± 0.23
GRA21	221.27 ± 3.34	211.93 ± 0.75	434.22 ± 3.08	241.17 ± 0.37	145.42 ± 0.28	386.59 ± 1.18	35.14 ± 0.45
GRA23	348.06 ± 4.24	189.41 ± 0.94	538.49 ± 2.23	138.95 ± 0.68	126.77 ± 0.65	265.72 ± 1.75	75.64 ± 0.38
GRA26	154.75 ± 2.08	68.71 ± 0.68	224.47 ± 3.16	115.88 ± 0.86	119.20 ± 0.32	235.08 ± 2.35	48.65 ± 0.74
GRA29	251.86 ± 3.15	50.72 ± 0.84	303.37 ± 4.08	69.15 ± 0.22	70.76 ± 0.27	139.91 ± 1.34	32.85 ± 0.68
Grand mean	239.10	115.14	555.24	115.02	122.01	277.03	45.74
F values	**	**	**	**	**	**	**
SE	1.252	1.016	1.126	1.612	0.931	1.412	0.756
CV%	3.523	1.246	1.452	2.025	2.147	1.465	2.258
CD	3.529	2.864	3.174	4.544	2.624	3.980	2.131

SE, Standard error difference; CV, Coefficient of variation; CD, Critical difference; n = 3; **Significant at 1% level.

The betacyanin content ranged from 69.15 to 241.17 ng g^−1^ FW with a mean value of 115.02 ng g^−1^ FW. The genotype GRA21 had the highest betacyanin content (241.17 ng g^−1^ FW), followed by that of GRA3 and GRA4. Conversely, the genotype GRA29 had the lowest betacyanin content (69.15 ng g^−1^ FW). Among the genotypes, significant variations were observed in the betaxanthin content, ranging from 70.76 to 164.29 ng g^−1^ FW. The betaxanthin content was the highest in the GRA9 genotype (164.29 ng g^−1^ FW) and relatively high in the GRA4, GRA7, GRA3, GRA15, GRA21, GRA23, and GRA26 genotypes. On the other hand, the genotype GRA29 showed the lowest betaxanthin content (70.79 ng g^−1^ FW). Seven genotypes had higher betaxanthin contents than the average content. The betalain content varied significantly and markedly and ranged from 139.91 to 392.82 ng g^−1^ FW. The genotype GRA4 had the highest betalain content (392.82 ng g^−1^ FW), and the genotypes GRA3, GRA9, and GRA21 had relatively high betalain contents. In contrast, the genotype GRA29 had the lowest content (139.91 ng g^−1^ FW). Five genotypes had higher betalain contents than the mean content of all genotypes. The carotenoid content ranged from 13.72 mg 100 g^−1^ FW in the GRA9 genotype to 88.44 mg 100 g^−1^ FW in GRA15. The genotype GRA15 showed the highest carotenoid content (88.44 mg 100 g^−1^ FW). The genotypes GRA7 and GRA23 had relatively high carotenoid contents. Five genotypes had higher carotenoid contents than the mean carotenoid content. In this study, we found remarkable chlorophyll *a* (239.10 μg g^−1^ FW), betaxanthin (122.01 ng g^−1^ FW), chlorophyll *b* (115.14 μg g^−1^ FW), betalain (277.03 ng g^−1^ FW), chlorophyll *ab* (555.24 μg g^−1^ FW), betacyanin (115.02 ng g^−1^ FW), and carotenoid (45.74 mg 100 g^−1^ FW) contents in the green morphs of amaranth. Khanam and Oba^41^ observed, more or less, similar trends in the betalain, chlorophyll *a*, carotenoid, chlorophyll *b*, β-xanthin, β-cyanin, and chlorophyll *ab* contents of green morph amaranth.

### Antioxidant phytochemicals and antioxidant capacity

The TAC, TPC, vitamin, and TFC values of the green morph amaranth are presented in Table 4. The vitamin C content ranged from 15.21 mg 100 g^−1^ FW in genotype GRA29 to 101.65 mg 100 g^−1^ FW in GRA3, with an average of 52.81 mg 100 g^−1^ FW. Six genotypes exhibited higher vitamin C contents than the corresponding mean content. The vitamin C content was relatively high in four genotypes, GRA4, GRA15, GRA21, and GRA26. The total polyphenol content (TPC) showed a wide range, with an average content of 14.83 GAE μg g^−1^ FW. The genotype GRA26 and GRA4 had the highest TPC than that of all other genotypes. Five genotypes had higher TPC values than the average value of all genotypes. The TFC exhibited noticeable variations among genotypes and ranged from 62.54 RE μg g^−1^ DW in the genotype GRA7 to 157.40 RE μg g^−1^ DW in GRA4. The mean value of TFC was 94.81 RE μg g^−1^ DW. The genotype GRA4 exhibited the highest TFC with the TFC order of GRA12 > GRA15 > GRA26 > GRA29. Five genotypes exhibited greater TFC values than the mean value of all genotypes. The TAC (DPPH) ranged from 8.90 TEAC μg g^−1^ DW (GRA26) to 26.56 TEAC μg g^−1^ DW (GRA4). The GRA29 genotype had high TAC (DPPH). In contrast, GRA26 had the lowest TAC (DPPH), with an average of 13.74 TEAC μg g^−1^ DW. Five genotypes had higher TAC values than the average value. The TAC (ABTS^+^) ranged from 19.16 TEAC μg g^−1^ DW (GRA17) to 48.12 TEAC μg g^−1^ DW (GRA4). The genotype GRA4 had the highest TAC (ABTS^+^). A relatively high TAC (ABTS^+^) was also observed in the GRA29 genotype. In contrast, the TAC (ABTS^+^) was the lowest in GRA17, with an average of 25.90 TEAC μg g^−1^ DW. Five genotypes exhibited higher TAC values than the overall mean value.

**Table 4:** Mean performance for vitamin C, TPC, TFC, TAC (DPPH) and TAC (ABTS^+^) in green morph *Amaranthus* leafy vegetable.

Genotypes	Vitamin C(mg 100 g^−1^ FW)	TPC (GAEµg g^−1^ FW)	TFC (REµg g^−1^ DW)	TAC (DPPH)(TEAC µg g^−1^ DW)	TAC (ABTS^+^)(TEACµg g^−1^ DW)
GRA3	101.65 ± 0.83	11.24 ± 0.05	85.27 ± 0.25	11.62 ± 0.12	22.24 ± 0.23
GRA4	67.69 ± 0.09	19.55 ± 0.04	157.40 ± 0.34	26.56 ± 0.13	48.12 ± 0.13
GRA7	36.89 ± 0.16	15.62 ± 0.05	62.54 ± 0.23	10.18 ± 0.04	18.36 ± 0.16
GRA9	58.53 ± 0.08	11.94 ± 0.06	64.40 ± 0.17	11.54 ± 0.12	24.08 ± 0.17
GRA11	18.87 ± 0.29	11.61 ± 0.04	75.64 ± 0.12	16.14 ± 0.13	28.28 ± 0.28
GRA12	42.36 ± 0.84	12.52 ± 0.02	125.64 ± 0.19	14.55 ± 0.10	26.10 ± 0.21
GRA15	65.69 ± 0.47	16.24 ± 0.92	108.31 ± 0.23	11.25 ± 0.12	21.50 ± 0.12
GRA17	45.25 ± 0.96	14.32 ± 0.08	76.37 ± 0.21	10.58 ± 0.14	19.16 ± 0.23
GRA21	67.85 ± 0.09	17.62 ± 0.05	84.77 ± 0.22	14.55 ± 0.12	28.10 ± 0.18
GRA23	49.06 ± 0.28	14.62 ± 0.08	68.00 ± 0.12	8.90 ± 0.14	19.80 ± 0.17
GRA26	64.69 ± 0.08	20.13 ± 0.04	124.38 ± 0.34	8.92 ± 0.11	16.84 ± 0.33
GRA29	15.21 ± 0.75	12.58 ± 0.02	104.98 ± 0.24	20.11 ± 0.12	38.22 ± 0.22
Grand mean	52.81	14.83	94.81	13.74	25.90
F values	**	**	**	**	**
SE	0.756	0.242	0.722	0.215	0.165
CV%	2.127	3.615	1.124	1.026	0.865
CD	2.131	0.682	2.035	0.606	0.465

SE, Standard error difference; CV, Coefficient of variation; CD, Critical difference; TAC = Total antioxidant capacity, TPC = Total polyphenol content, TFC = Total flavonoid content, n = 3; **Significant at 1% level.

In this study, we found a remarkable vitamin C content (101.65 mg 100 g^−1^ FW) in the green morphs of amaranth, which was higher than our earlier results in red morphs of amaranth^6^. The TPC values (20.13 GAE μg g^−1^ FW) obtained in this study were higher than the results of Khanam *et al*.^42^ in green morphs of amaranth. The TAC (ABTS^+^) (48.12 TEAC μg g^−1^ DW), TFC (157.40 RE μg g^−1^ DW), and TAC (DPPH) (26.56 TEAC μg g^−1^ DW) values obtained in this study for green morph amaranth showed a similar trend to the results of Khanam *et al*.^42^. The genotypes GRA4, GRA11, GRA12, and GRA21 had high phenolic and antioxidant vitamin contents along with high TAC values. These genotypes could be used as high-yielding antioxidant-rich varieties. Our results have shown that green amaranth is abundant in antioxidant phytochemicals, proximate composition, antioxidant activity, and minerals and offers a large possibility of supplying minerals, vitamins, and antioxidants to deficient communities.

### Correlation coefficient analysis

The correlations of TAC (DPPH), TFC, antioxidant leaf pigments, TAC (ABTS^+^), vitamin C, and TPC of green amaranth are presented in Table 5. The above mentioned correlations showed interesting results. All antioxidant leaf pigments exhibited significant positive associations with TAC (ABTS^+^), TFC, TAC (DPPH), and TPC. This signifies that the increases in carotenoids, betaxanthins, chlorophyll, betacyanins, and betalains were directly related to the increases in TAC (ABTS^+^), TPC, TAC (DPPH), and TFC.

**Table 5:** Correlation co-efficient for antioxidant leaf pigments, vitamin C, TPC, TFC, TAC (DPPH) and TAC (ABTS^+^) in green morph *Amaranthus* leafy vegetable.

Traits	Chl *b* (µg g^−1^ FW)	Chl *ab* (µg g^−1^ FW)	Betacyanin (ng g^−1^ FW)	Betaxanthin (ng g^−1^ FW)	Betalain (ng g^−1^ FW)	Catonenoirds (mg 100 g^−1^ FW)	Vitamin C (mg 100 g^−1^ FW)	TPC(GAEµg g^−1^ FW)	TFC (RE µg g^−1^ DW)	TAC (DPPH) (TEAC µg g^−1^ DW)	TAC (ABTS^+^) (TEAC µg g^−1^ DW)
Chlorophyll *a* (µg g^−1^ FW)	0.95**	0.87**	0.76**	0.78**	0.78**	−0.56**	−0.002	0.47**	0.47**	0.68**	0.66**
Chlorophyll *b*(µg g^−1^ FW)		0.88**	0.66**	0.65**	0.66**	−0.58**	−0.014	0.49**	0.58**	0.68**	0.64**
Chlorophyll *ab* (µg g^−1^ FW)			0.78**	0.74**	0.68**	−0.62**	−0.005	0.58**	0.55**	0.65**	0.63**
Betacyanin (ng g^−1^ FW)				0.89**	0.85**	−0.79**	−0.106	0.55**	0.56**	0.73**	0.75**
Betaxanthin (ng g^−1^ FW)					0.98**	−0.65**	−0.123	0.68**	0.59**	0.69**	0.78**
Betalain (ng g^−1^ FW)						−0.78**	−0.141	0.68**	0.68**	0.78**	0.73**
Catonenoirds (mg 100 g^−1^ FW)							−0.183	0.54**	0.55**	0.75**	0.85**
Vitamin C (mg 100 g^−1^ FW)								0.08	0.08	0.08	0.009
TPC (GAE µg g^−1^ FW)									0.75**	0.66**	0.96**
TFC (RE µg g^−1^ DW)										0.75**	0.88**
TAC (DPPH) (TEAC µg g^−1^ DW)											0.94**

Chl *a*, Chlorophyll *a;* Chl *ab*, Chlorophyl *ab;* TAC, Total antioxidant capacity; TPC, Total polyphenol content; TFC, Total flavonoid content; **Significant at 1% level.

Similarly, vitamin C had a minor and positive interrelationship with TAC, TFC, and TPC, although it exhibited negative and insignificant associations with all pigments. In their earlier work on red amaranth, Shukla *et al*.^10^; Sarker and Oba^23^ reported corroborative results similar to those of our present study. TAC (ABTS^+^), TPC, TAC (DPPH), and TFC showed a significant positive association. These results indicate that TFC and TPC have strong antioxidant activity. Similarly, the significant interrelationship between TAC (ABTS^+^) and TAC (DPPH) validated the antioxidant capacity of green amaranth by two different methods of estimation. The phenolic compounds, vitamin C, antioxidant pigments, and flavonoids had strong antioxidant activity. In the present investigation, it was revealed that flavonoids, phenolic compounds, and all pigments had strong antioxidant activity and significantly contributed to the antioxidant activity of the green morphs of amaranth.

The leaves of the green morph amaranth were good sources of K, Ca, Mg, iron, manganese, copper, zinc, protein, dietary fiber, carbohydrates, antioxidant leaf pigments, vitamin C, phenolic compounds, flavonoids, and antioxidants. Thus, amaranth could be used as a leafy vegetable for potential sources of phenolics, macronutrients, flavonoids, pigments, vitamin C, minerals, and antioxidants in our daily diet to combat hidden hunger and attain nutritional and antioxidant sufficiency. Therefore, staple foods (e.g., rice, wheat, maize) combined with the leaves of green morph leafy amaranth could contribute to reducing the incidence of hidden hunger worldwide.

## Methods

### Experimental materials, design, layout, and cultural practices

Twelve distinct and promising genotypes of green morph amaranth from our previous collection were grown at Bangabandhu Sheikh Mujibur Rahman Agricultural University, Bangladesh. The experiment was carried out following a randomized complete block design (RCBD) in three replicates. Each experimental unit was one square meter. Green morph amaranth genotypes were grown maintaining 20 cm between rows and 5 cm between plants. The experimental field was high land with silty clay soil. The soil was slightly acidic (pH 6.4) and low in organic matter (0.87%), total N (0.09%), and exchangeable K (0.13 cmol/kg). The soil S content was near the critical level, while the P and Zn contents were above the critical level (critical levels of P, S, and Zn were 14, 14 and 0.2 mg kg^−1^, respectively, and that of K was 0.2 cmol kg^−1^). During land preparation, a total compost (10 ton/ha) was applied. We maintained recommended fertilizer doses, such as urea, triple superphosphate, murate potash and gypsum concentrations of 200, 100, 150, and 30 kg/ha, respectively. Thinning, weeding and hoeing were performed whenever necessary. Proper irrigation was provided to maintain the normal growth of the crop. Leave samples were collected 30 days after seed sowing. All parameters were measured in three replicates.

### Chemicals

Solvents: acetone and methanol. Reagents: H_2_SO_4_, HNO_3_, HClO_3_, NaOH, dithiothreitol (DTT), cesium chloride, ascorbic acid, standard compounds of pure Trolox (6-hydroxy-2, 5, 7,8-tetramethyl-chroman-2-carboxylic acid), gallic acid, rutin, folin-ciocalteu reagent, DPPH (2,2-diphenyl1-picrylhydrazyl), ABTS^+^, aluminum chloride hexahydrate, sodium carbonate, potassium acetate, and potassium persulfate. All solvents and reagents were purchased from Merck (Germany) and Kanto Chemical Co. Inc. (Tokyo, Japan).

### Estimation of proximate composition

ASAE standards^43^ were followed to measure the moisture content of the samples. Green morph *Amaranthus* leaf samples (triplicate) were oven-dried at 103 °C for 72 h, transferred to a desiccator, and allowed to cool at room temperature. A digital balance (Denver Instruments, Denver, Colorado, USA) was used to record the sample weights.

AOAC methods^44^ were used to determine ash, crude fat, and crude protein contents. The leaf samples were weighed before and after heat treatment (550 °C for 12 h), and the ash content was determined. AOAC method 960.39 was used to determine the crude fat content.

The micro-Kjeldahl method was followed to determine crude protein, multiplying nitrogen by 6.25 (AOAC method 976.05). The ISO method 5498:1981 method^45^ was used to determine dietary fiber content. Green morph *Amaranthus* leaf samples were boiled in 0.255 M sulfuric acid for 30 min. Exactly 0.313 M sodium hydroxide was used to filter, wash, and boil the resulting insoluble residue. Finally, the residue was dried at 130 ± 2 °C for 2 h. Weight loss was determined at 350 ± 25 °C. The dietary fiber content was expressed as g 100 g^−1^ FW.

The sum of the percentage of crude protein, moisture, crude fat, and ash contents was subtracted from 100 to estimate the carbohydrate content (g kg^−1^ FW). ISO 9831 method^46^ was used to determine the gross energy content using a bomb calorimeter.

### Determination of mineral composition

Leaves of green amaranth were dried at 70 °C in an oven for 24 h. Dried leaves were grounded finely in a mill. An 841 micron screen was used to separate finely milled powder. The final dried and milled powder was analyzed for macronutrients (calcium, magnesium, and potassium) and microelements (copper, iron, zinc, and manganese). The nitric-perchloric acid digestion method^47^ was followed to determine calcium, potassium, magnesium, iron, manganese, copper, and zinc from powdered leaves. For this digestion, in the presence of carborundum beads, 40 ml HClO_4_ (70%), 400 ml HNO_3_ (65%), and 10 ml H_2_SO_4_ (96%) were added to a 0.5 g dried leaf sample. After digestion, the solution was appropriately diluted in triplicate for measuring P following the ascorbic acid method. The addition of ascorbic acid and Sb to the yellow-colored complex solution converted it to a blue-colored phosphomolybdenum complex. According to the method of Temminghoff and Houba^48^, the absorbance was determined by atomic absorption spectroscopy (AAS) (Hitachi, Tokyo, Japan) at wavelengths of 285.2 nm (magnesium), 766.5 nm (potassium), 248.3 nm (iron), 422.7 nm (calcium), 279.5 nm (manganese), 213.9 nm (zinc), and 324.8 nm (copper). AAS standard solutions (1000 mg l^−1^ in 5% HNO_3_) (Merck, Germany) were used for calibration. Finally, lanthanum and cesium chloride (0.1%) were added to the samples and standards for mitigating interferences.

### Estimation of chlorophyll and carotenoid contents

The leaves of green amaranth were extracted in 80% acetone to estimate the chlorophyll *ab*, chlorophyll *b*, total carotenoid, and chlorophyll *a* contents according to the method of Lichtenthaler and Wellburn^49^. A spectrophotometer (Hitachi, U-1800, Tokyo, Japan) was used to read the absorbance at 663 nm for chlorophyll *a*, 646 nm for chlorophyll *b*, and 470 nm for total carotenoids. Data were expressed as μg chlorophyll per g fresh weight (FW) and mg carotenoids per 100 g FW.

### Estimation of betacyanin and betaxanthin content

The leaves of green amaranth were extracted in 80% methyl alcohol with 50 mM ascorbate to measure the betacyanin and betaxanthin contents according to the method of Wyler *et al*.^50^. A spectrophotometer (Hitachi, U-1800, Tokyo, Japan) was used to measure the absorbance at 540 nm for betacyanins and 475 nm for betaxanthins. Mean molar extinction coefficients (62 × 10^6^ cm^2^ mol^−1^ for β-cyanin and 48 × 10^6^ cm^2^ mol^−1^ for β-xanthin) were used to quantify the betacyanins and betaxanthins. The results were expressed as nanograms betanin equivalent per gram FW for betacyanins and nanograms indicaxanthin equivalent per gram FW for betaxanthins.

### Estimation of vitamin C

Fresh green amaranth leaves were used to measure ascorbate (AsA) and dehydroascorbic acid (DHA) acid with a spectrophotometer. For preincubation of the samples and reduction of DHA into AsA, dithiothreitol (DTT) was used. AsA reduced Fe_3_^+^ to Fe_2_^+^, and estimation of AsA was made by spectrophotometry (Hitachi, U-1800, Tokyo, Japan) measuring Fe_2_^+^ complexes with 2,2-dipyridyl^51^. Finally, the absorbance of the sample solution was read. Data were recorded as mg ascorbic acid per 100 g fresh weight (FW).

### Sample extraction for TFC, TAC, and TPC analysis

At 30 DAS, green amaranth leaves were harvested. For chemical analysis, the leaves were dried in the air in a shade. 40 ml 90% aqueous methanol was used to extract 1 g of ground, dried leaves from each cultivar in a bottle (100 ml) capped tightly. A shaking water bath (Thomastant T-N22S, Thomas Kagaku Co. Ltd., Japan) was used to the extract the samples for 1 h. The extracts were filtered for the determination of polyphenols, flavonoids, and total antioxidant capacity.

### Estimation of polyphenols

The method of Velioglu *et al*.^52^ was followed to estimate the total phenolic content of green amaranth using the Folin-Ciocalteu reagent with gallic acid as a standard phenolic compound. Folin-Ciocalteu reagent was previously diluted to 1:4 reagent:distilled water. In a test tube, 1 ml diluted Folin-Ciocalteu reagent was added to 50 µl extract solution and then mixed thoroughly for 3 min. Then, 1 ml of Na_2_CO_3_ (10%) was added to the tube and incubated for 1 h in the dark. A Hitachi U1800 spectrophotometer (Hitachi, Tokyo, Japan) was used to read the absorbance at 760 nm. Total phenolic compounds in the green amaranth leaf extracts were determined using an equation obtained from a standard gallic acid graph with gallic acid concentrations on the X-axis and their corresponding absorbance values on the Y-axis. The results are expressed as μg gallic acid equivalent (GAE) g^−1^ FW.

### Estimation of flavonoids

The AlCl_3_ colorimetric method outlined by Chang *et al*.^53^ was used to estimate the total flavonoid content of green amaranth extracts. In a test tube, 1.5 ml methanol was added to 0.1 ml 10% aluminum chloride, 0.1 ml 1 M potassium acetate, 2.8 ml distilled water and 500 µl leaf extract for 30 min at room temperature. A Hitachi U1800 spectrophotometer (Hitachi, Tokyo, Japan) was used to read the absorbance of the reaction mixtures at 415 nm. TFC was expressed as μg rutin equivalent (RE) g^−1^ dry weight (DW) using rutin as the standard compound.

### Estimation of antioxidant capacity (TAC)

The diphenyl-picrylhydrazyl (DPPH) radical degradation method^54^ was used to estimate the antioxidant activity of the amaranth extracts. In a test tube, 1 ml 250 µM DPPH solution was added to 10 µl leaf extract solution (in triplicate) and 4 ml distilled water and allowed to stand for 30 min in the dark. A Hitachi U1800 spectrophotometer (Hitachi, Tokyo, Japan) was used to read the absorbance at 517 nm. The method of Thaipong *et al*.^55^ was followed for the ABTS^+^ assay. A 7.4 mM ABTS^+^ solution and 2.6 mM potassium persulfate were used as the stock solutions. The two stock solutions were mixed in equal quantities and allowed to react for 12 h at room temperature in the dark for preparation of the working solution. A total of 2850 μl ABTS^+^ solution (1 ml ABTS^+^ solution mixed with 60 ml methanol) was mixed with 150 μl leaf extract and allowed to react for 2 h in the dark. A Hitachi U1800 spectrophotometer (Hitachi, Tokyo, Japan) was used to read the absorbance against methanol at 734 nm. The percent inhibition of DPPH and ABTS^+^ relative to the control was used to determine antioxidant activity using the following equation:

$${\rm{Antioxidant}}\,{\rm{activity}}\,( \% )=({\rm{Abs}}.\,{\rm{blank}}-{\rm{Abs}}.\,{\rm{sample}}/{\rm{Abs}}.\,{\rm{blank}})\times 100$$

where Abs. blank is the absorbance of the control reaction [10 µl methanol for TAC (DPPH), 150 μl methanol for TAC (ABTS^+^) instead of leaf extract] and Abs. sample is the absorbance of the test compound. Trolox was used as the reference standard, and the results were expressed as μg Trolox equivalent g^−1^ DW.

### Statistical analysis

Replicate samples were averaged to obtain replication means. Mean data of triplicate samples were also statistically analyzed by ANOVA using Statistix 8 software, and the means were compared by Tukey’s HSD test at a 1% level of probability. The results were reported as the average of three replications ± SD.

### Ethical statement

The lab and field experiments in this study were carried out as per guidelines and recommendations of “Biosafety Guidelines of Bangladesh” published by the Ministry of Environment and Forest, Government of the People’s Republic of Bangladesh (2005).

## Data Availability

Data used in this manuscript will be available to the public.
